# CASS: A distributed network clustering algorithm based on structure similarity for large-scale network

**DOI:** 10.1371/journal.pone.0203670

**Published:** 2018-10-10

**Authors:** Jungrim Kim, Mincheol Shin, Jeongwoo Kim, Chihyun Park, Sujin Lee, Jaemin Woo, Hyerim Kim, Dongmin Seo, Seokjong Yu, Sanghyun Park

**Affiliations:** 1 Department of Computer Science, Yonsei University, Seoul, South Korea; 2 Korea Institute of Science and Technology Information, Daejeon, South Korea; Texas A&M University College Station, UNITED STATES

## Abstract

As the size of networks increases, it is becoming important to analyze large-scale network data. A network clustering algorithm is useful for analysis of network data. Conventional network clustering algorithms in a single machine environment rather than a parallel machine environment are actively being researched. However, these algorithms cannot analyze large-scale network data because of memory size issues. As a solution, we propose a network clustering algorithm for large-scale network data analysis using Apache Spark by changing the paradigm of the conventional clustering algorithm to improve its efficiency in the Apache Spark environment. We also apply optimization approaches such as Bloom filter and shuffle selection to reduce memory usage and execution time. By evaluating our proposed algorithm based on an average normalized cut, we confirmed that the algorithm can analyze diverse large-scale network datasets such as biological, co-authorship, internet topology and social networks. Experimental results show that the proposed algorithm can develop more accurate clusters than comparative algorithms with less memory usage. Furthermore, we confirm the proposed optimization approaches and the scalability of the proposed algorithm. In addition, we validate that clusters found from the proposed algorithm can represent biologically meaningful functions.

## Introduction

A network is a useful data structure for quickly and efficiently managing data. It additionally inherently includes several features that can be analyzed, such as clustering, shortest path, degree, and propagation. Of these, clustering is widely used to analyze network data in several research areas. In biology, for example, a network is used to describe complex relationships between biological entities. Moreover, network clustering is an important analysis algorithm because the groups, which are inferred from the clustering results, enable the opportunity to understand the biological relationships between nodes that are included in the same cluster. Zhang et al. [1], for example, attempted to identify functional modules in a protein–protein network (PPI) using network clustering. They demonstrated that network clustering can be used to identify important interactions in biological networks. Jones et al. [2] presented a method to analyze social networks using a clustering algorithm. The proposed algorithm is based on the SCAN algorithm. SCAN is a well-known conventional network clustering algorithm. The algorithm is based on structure similarity, which is calculated by the number of common neighborhoods between nodes. Using SCAN, Jones et al. identified the hub and outlier nodes in a social network. In their approach, the hub indicates that nodes have connections with many clusters, and outliers indicate nodes that are not included in clusters and have no connections with them. By inferring the hub and outlier nodes, they identified key members and the less important members in a given social network. Their results showed that clustering is a useful approach to analyze social networks. Network clustering is additionally used in sensor network analysis to consider energy consumption. Cheng et al. [3] attempted to extend the lifetime of a wireless sensor network using a clustering technique. They presented a low-energy clustering network architecture to prolong wireless network lifetime.

Conventional clustering algorithms, however, are generally only implemented in a single machine environment, not in distributed systems. As the amount of data is rapidly increasing in modern times, conventional algorithms cannot analyze this type of large-scale network data because of inherent memory problems or need huge computation time. In addition, many algorithms are not suitable for operation in a distributed system. For example, van Dongen [4] proposed the Markov Clustering Algorithm (MCL) based on a flow matrix. MCL derives an initial flow matrix from an adjacency matrix and then finds clusters by repeating update, inflate, and prune operations until the flow matrix converges. MCL is a simple and intuitive algorithm. However, because it detects clusters through matrix operations, memory resources are required to store the entire adjacency matrix; therefore, it is not suitable for operation in a distributed system environment.

To solve this problem, several studies applied the MapReduce programming model [5] for large-scale network analysis [6, 7]. The MapReduce programming model is a renowned, widely used programming model to process big data with a parallel and distributed algorithm. This programming model processes a large dataset through a map function and a reduce function. The map function converts the input dataset into key-value pairs, while the reduce function aggregates and processes the key-value pairs generated by the map function. Shiokawa et al. [8] proposed a novel data structure to reduce the execution time for big data network clustering using the MapReduce model.

The MapReduce model supported by Hadoop is a model that offers a way which is easy and efficient to parallelize large computations on many computers. The Hadoop MapReduce-based clustering algorithm was presented by Zhao et al. [9]. They proposed the PSCAN method, which is a parallel structure clustering algorithm for big-data networks using the Hadoop MapReduce model. They modified SCAN to become a parallel algorithm for processing big data. The algorithm has two main functions: map and reduce. The map function refines the adjacency list of the vertex, while the reduce function calculates structure similarity. Experimental results showed that their algorithm can cover big network data with many nodes and edges. However, the Hadoop MapReduce model must store intermediate results into a distributed file system (DFS) for each iteration, so it is an unsuitable model for network clustering algorithms that include iterative tasks. This challenge can be solved by employing the Apache Spark framework. Apache Spark [10, 11] overcomes the drawbacks of the Hadoop MapReduce model by adapting new features, specifically a resilient distributed dataset (RDD), a broadcast variable, and extensible operation units. Spark does not store processed data on disks. Most data reside in volatile memory and are immutable. This data is called RDD. RDD is transformed into new RDD, and the previous RDD is not modified and is simply released from memory. This concept greatly increases the speed of iterative mining algorithms. For this reason, several iterative mining algorithms have been designed to process big data using the Spark framework [12, 13, 14, 15, 16]. In network clustering algorithms, Matteo et al. [17] proposed a k-center clustering algorithm that locates disjoint connected clusters by gradually growing clusters from batches of centers using Apache Spark. They demonstrated through experimental results that the proposed algorithm has two advantages. First, it is easy to control the number of clusters and the maximum radius of the clusters, and second, it only requires linear space computations for the given problem size. However, the k-center algorithm does not consider network structure and therefore, it is difficult to expect good performance when analyzing a network that needs to consider the structure. Another problem is that most network clustering algorithms operating in distributed system environments are not optimized. For example, as the size of the network data to be analyzed increases, the data generated in the analysis process also significantly increases. These factors can have a significant impact on the performance of the algorithm, but there are few studies related to this.

In this study, we propose a new distributed network Clustering Algorithm based on Structure Similarity (CASS) for large-scale networks in the Apache Spark environment. The proposed algorithm uses structure similarity [18] to calculate the weights of edges. Structure similarity is a score that can represent the similarity between two different nodes. The higher the number of adjacent nodes shared by two nodes, the higher the score. Because the calculation of structure similarity represents a large portion of the computational complexity of the algorithm, we changed this process in a form suitable for Apache Spark by using a triangle structure and join operations. Furthermore, we employ optimization approaches such as Bloom filter and shuffle selection to improve the proposed algorithm, and we analyze clusters found from Humannet to validate whether the proposed algorithm can find biologically significant clusters. The main contributions of this work are as follows:

1. Developing a clustering algorithm for large-scale networks in distributed systems.
2. Changing the process of calculating structure similarity to a form suitable for distributed system environments using a triangle structure and join operations.
3. Reducing the execution time by considering optimization approaches.

## Materials and methods

### Data description

In this study, we used various types of undirected networks to evaluate the performance of the proposed algorithm. The characteristics of these networks are summarized in Table 1.

**Table 1:** Characteristics of the datasets.

Name	# Node	# Edge	Diameter	Description
Drosophila	6,600	19,820	11	Drosophila protein interaction network
DIP	22,585	69,148	25	Protein interaction network
DBLP	317,080	1,049,866	21	Co-authorship network
As-skitter	1,696,415	11,095,298	25	Internet topology network
Com-LiveJournal	3,397,962	34,681,189	17	LiveJournal online social network
Humannet	476,399	16,243	10	Probabilistic functional gene network of homo sapiens

In the table, ‘Name’ is the name of the database. ‘# Node’ is the number of nodes and ‘# Edge’ is the number of edges. ‘Diameter’ indicates the longest shortest path in the network. Drosophila was downloaded from [19] and Humannet v.1 was downloaded from [20]. DIP was downloaded from the Interologous Interaction Database [21] and the remaining datasets were downloaded from SNAP [22].

### Overview of proposed algorithm

**Fig 1:**
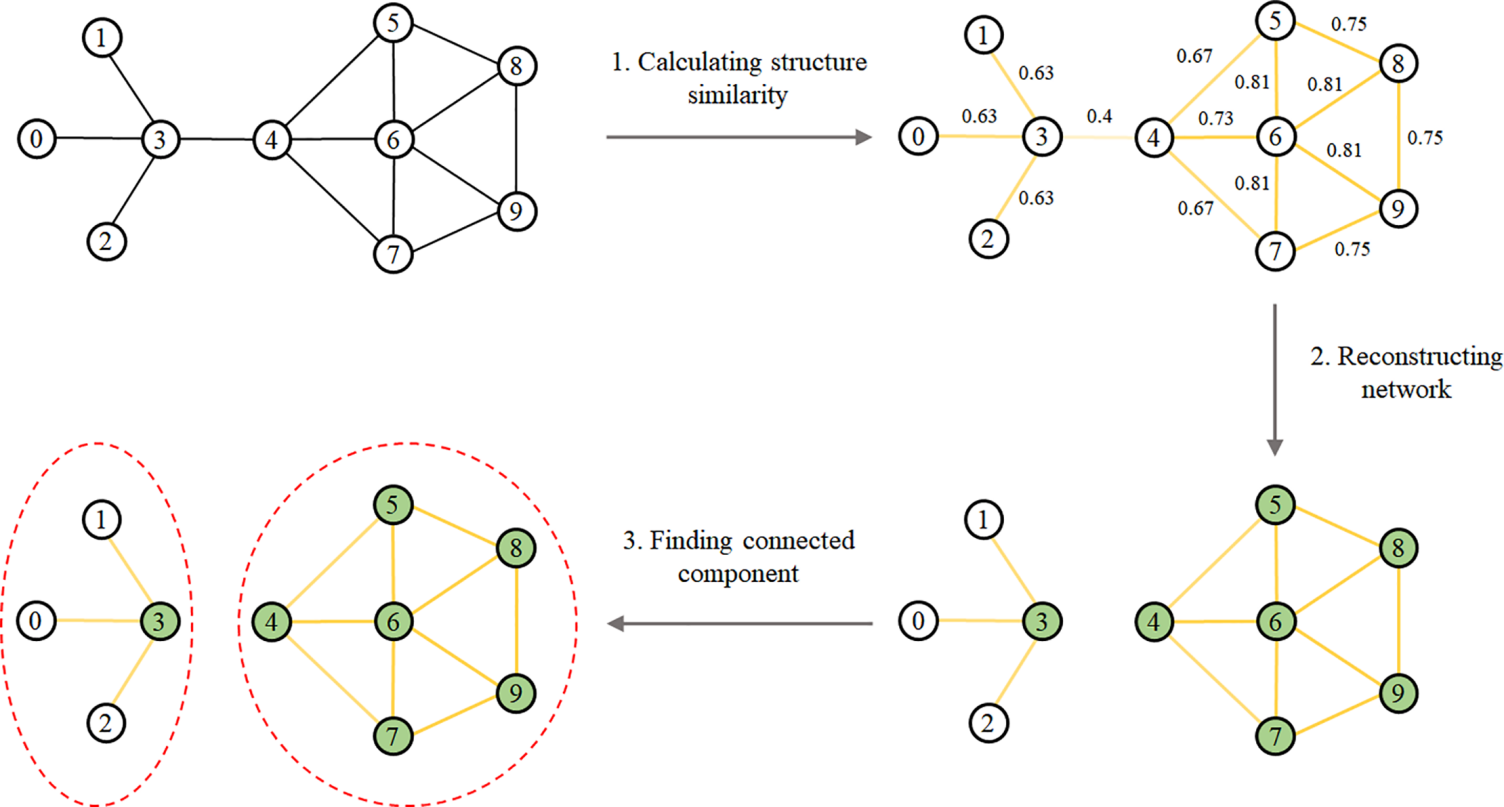
Overview of CASS. (m = 2, e = 0.6).

Fig 1 shows an overview of the proposed algorithm. In the first step, we calculate structure similarity between nodes. Structure similarity is a measure indicating the degree of similarity between two nodes. It can be calculated from the number of adjacent nodes shared by two different nodes. In the above figure, w represents the structure similarity, and the edge contrast represents the degree of structure similarity. In the next step, the edges of the network are refined based on the user-configured epsilon value (= 0.6). Then, each node is labeled as core or non-core. More specifically, if a node has more adjacent nodes than the m value configured by the user, the node is labeled as core, otherwise it is labeled as non-core. We then refine the edges of the network once more based on the label of each node. In the figure, the filled (colored) nodes indicate a core, and the uncolored nodes indicate a non-core. In the final step, connected components are found, and each connected component is considered a cluster.

### Calculating structure similarity based on triangle structure

In this section, we introduce the manner in which we calculate the structure similarity based on the triangle structure. Structure similarity is a measure of the similarity between two different nodes, and the larger the number of adjacent nodes shared by the two nodes, the larger the similarity. The equation to obtain structure similarity is as follows.

$$\begin{array}{c} Structure\;similarity(v,w)=\frac{\left|Nei(v)\cap Nei(w)\right|}{\sqrt{\left|Nei(v)||Nei(w)\right|}}\\ \;V:The\;vertex\;set\;of\;a\;graph\\ \;E:The\;edge\;set\;of\;a\;graph\\ Nei(v)=\{w\in V|(v,w)\in E\}\cup \{v\} \end{array}$$

In the equation, Structure similarity (v,w) is the structure similarity between nodes *v* and *w* and has a value between 0 and 1. As shown in the above equation, it is necessary to obtain all adjacent nodes of each node to calculate structure similarity. In this study, we find adjacent nodes using the triangle structure.

**Fig 2:**
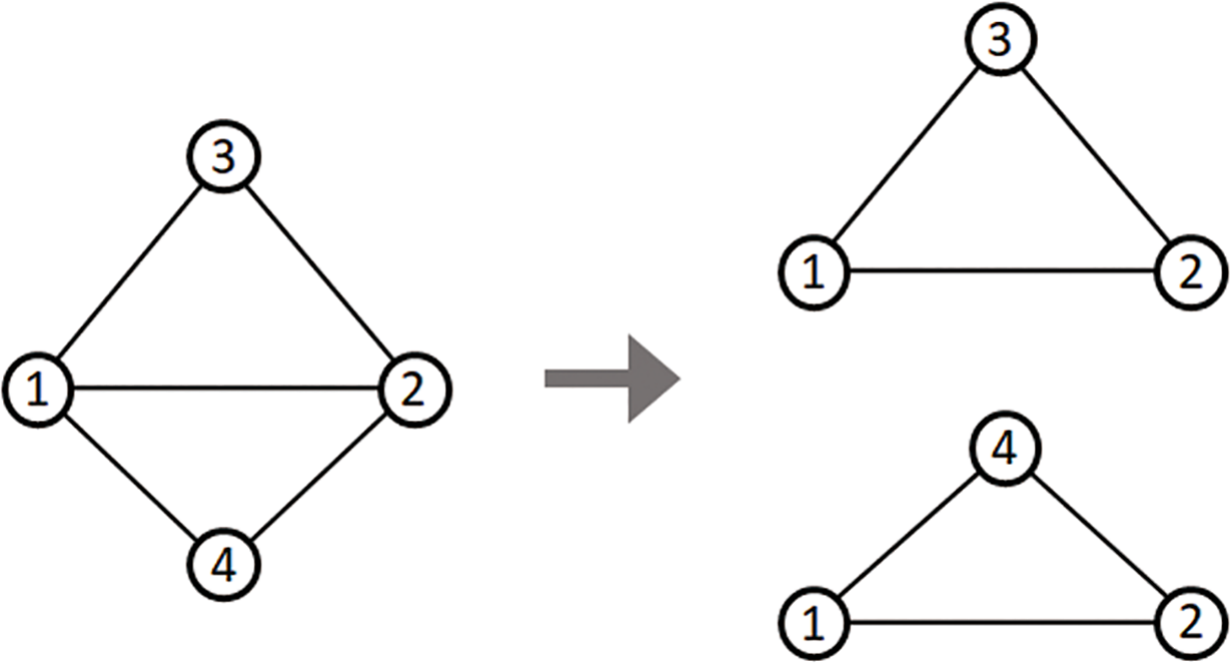
Example of the triangle structure.

Fig 2 shows the principle for finding adjacent nodes of two different nodes using the triangle structure. As shown the figure, the problem of finding adjacent nodes that are shared by node ‘1’ and node ‘2’ can be replaced with the problem of finding the number of triangles with node ‘1’ and node ‘2’ as one side. Therefore, in this study, we find all triangles in the graph and use these to calculate the number of adjacent nodes shared by two different nodes.

**Fig 3:**
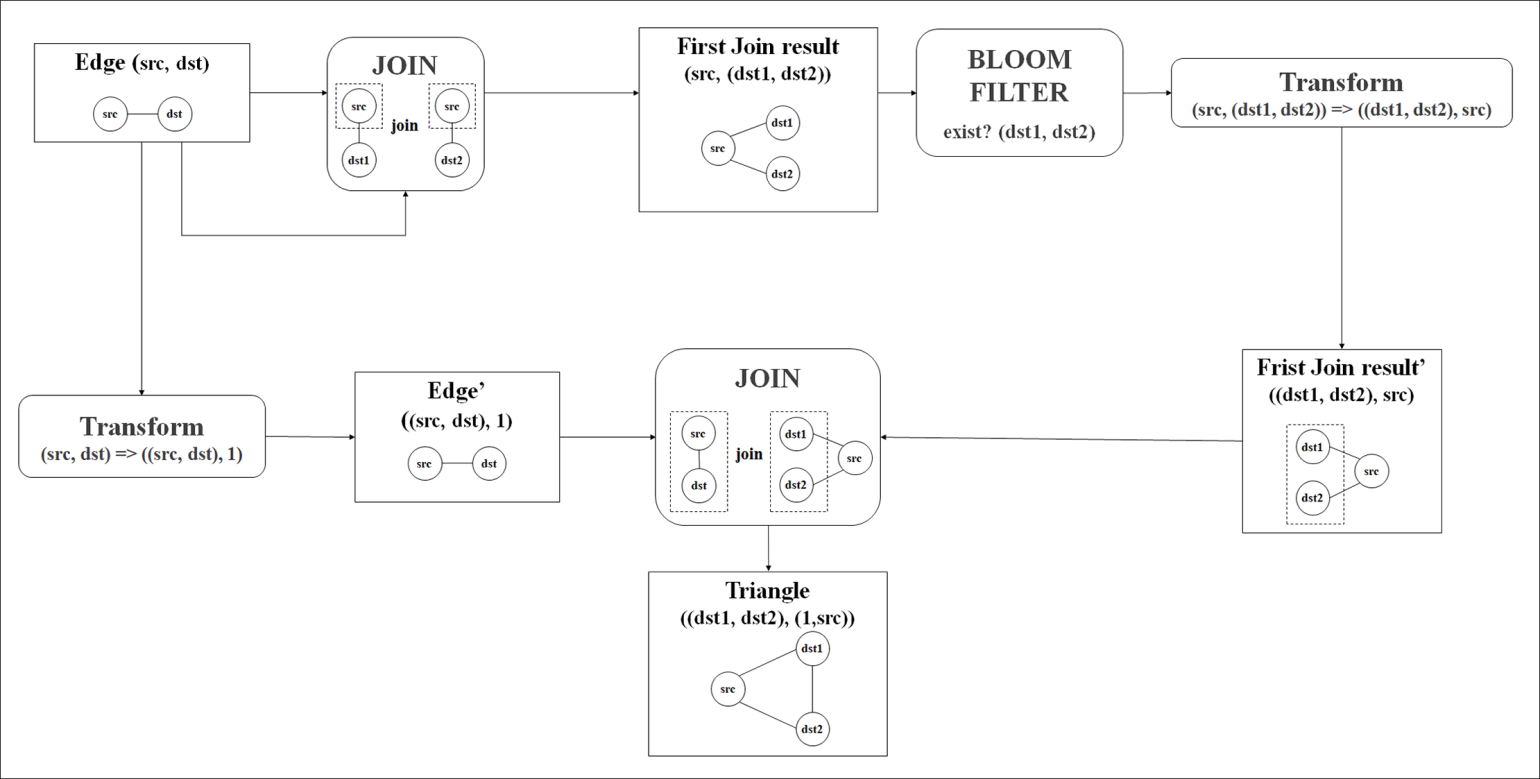
Structure similarity calculation.

Fig 3 presents the process of the structure similarity calculation in Spark. The number of triangle structures is calculated through two join operations. The data consist of a key-value structure in Spark. In the round brackets, the first part is the key and the second part is the value. First, we conduct the join operation for edge data. By joining the edge data, we obtain a candidate triangle structure, which has two edges and three nodes. To obtain the triangle structure, we conduct the join operation between the reversed data for the candidate triangle structure and data, which consists of an edge and a “1.” The “1” is only used to create the data structure. As a result, we can locate the triangle structure using the edge data and join operations. Using this structure and edge information, we calculate the structure similarity for all edges. The Bloom filter, which is used for optimization, is described in Section 2.6.

### Network re-constructing

After calculating the structure similarity, we prune the edges whose structure similarity value is lower than the user-configured ‘e’ value. Then, we assign a core or non-core label to each node. If a node has more adjacent nodes than the user-specified ‘m’ value, the node is labeled as core; otherwise it is labeled as non-core. After assigning labels to all nodes, we refine edges based on the labels. The rules of refining are:

- The adjacent nodes of a core node are preserved whether adjacent nodes are core nodes or not.
- The adjacent nodes of a non-core node are preserved if the adjacent nodes of a non-core node are core nodes.
- The adjacent nodes of a non-core node are pruned if the adjacent nodes of a non-core node are non-core nodes.

As a result, only the edges between core nodes and the edges between core and non-core nodes remain.

### Connected components

In this section, we locate clusters from the reconstructed network. In other words, we find clusters by assigning all the adjacent nodes of a core-node to the same cluster. Because we removed all edges between non-cores in the previous step, we can locate the desired clusters by searching for connected components. A connected component is a set of nodes in a network which are connected to each other by paths. In our study, we considered these connected components as clusters.

**Fig 4:**
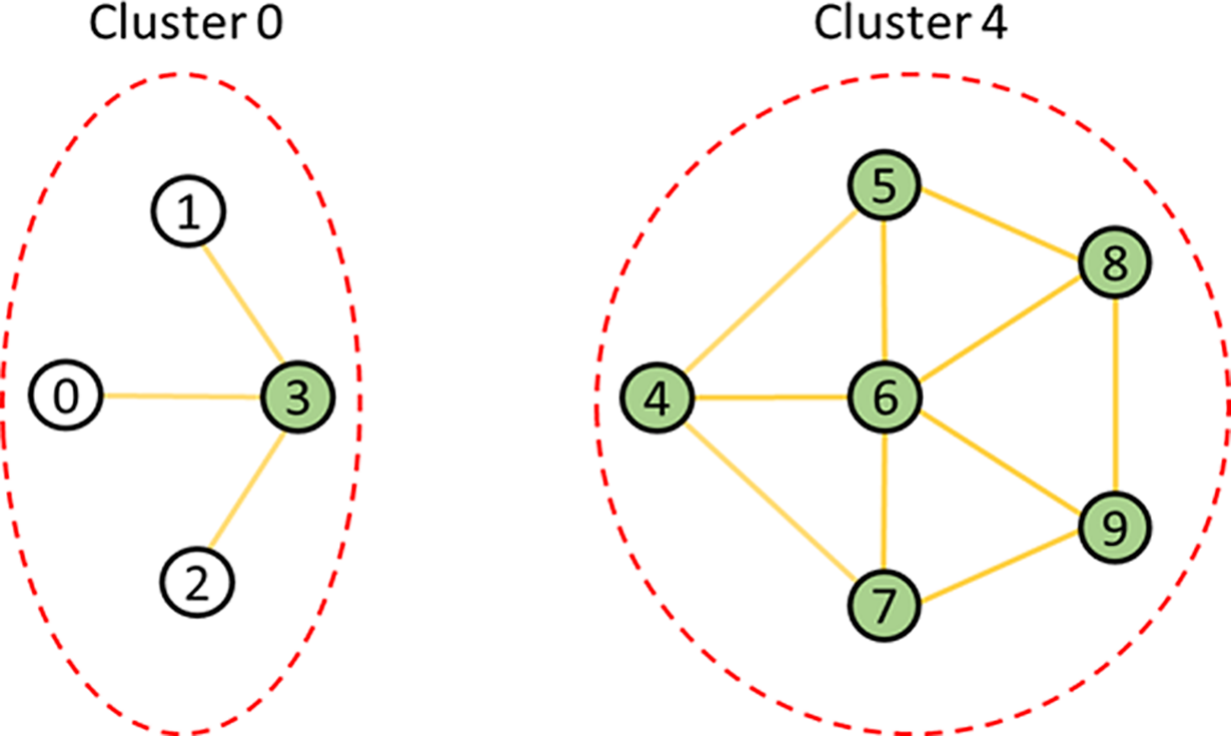
Examples of clusters.

Fig 4 shows example clusters. In the figure, the red circle drawn as a dotted line represents one cluster, and the lowest number among the nodes in the cluster becomes the cluster identifier. For example, in the figure, the cluster identifier on the left cluster is 0 because the node numbered 0 in the left cluster has the lowest number. Also, there are no shared edges between two different clusters.

### Optimization

In this section, we address optimization for reducing computation time. The proposed algorithm has two representative optimizations which include the Bloom filter and shuffle selection.

#### Bloom filter

As shown in Fig 3, finding triangles requires two join operations with an edge RDD, and the results generated can be extremely large. The first join operation generates information about one vertex and two adjacent nodes connected with the vertex. The second join operation checks whether two adjacent nodes are connected. To perform the second join operation, the key of the RDD joined by the first join operation should be changed from the common vertex to the edge composed of two adjacent nodes. Changing the key causes repartitioning and shuffling of the first-joined RDD through the network.

To reduce shuffling of the intermediate results, we employ the Bloom filter [23], which enables verification of the existence of each edge. The Bloom filter is a probabilistic data structure which is used to check whether an element possibly belongs to a set or definitely does not belong to a set. The filter uses a bit size array and hash functions to do this. For example, when an element is added to the Bloom filter, the filter calculates hash values for the input element, and then sets the bit corresponding to each hash value to bit array as 1. Then, when an element is checked, hash values are calculated for the element that has to be checked, and then the bit value corresponding to each hash value is read. If all the bits are 1, it is judged to belong to the data set. The Bloom filter has a feature that if the filter determines that an element may belong to a set, it is possible to generate a false positive error. On the other hand, when an element is determined not to belong to a set, a false negative error never occurs. Therefore, if we check the existence of two adjacent nodes after the first join operation using the Bloom filter, the intermediate result can be greatly reduced. For example, suppose that 500 intermediate results are generated as a result of a first join, and 100 of them are passed through a Bloom filter with a false-positive ratio of 10%. In this case, if the edge size of the network is 200, 100,000 (500 * 200) operations in the second join can be reduced to 20,000 (100 * 200) operations. Now, the unnecessary join operations are 2,000 (10 * 200) operations.

After an RDD is created for edges, our algorithm builds a Bloom filter whose key is each edge. Our Bloom filter works as follows:

$$bf(e)=\left\{ \begin{array}{c} edge\;does\;not\;exist\\ edge\;can\;exist \end{array}\right.$$

Above, *bf* is the Bloom filter and *e* is an edge to be checked. Our algorithm filters the key-value pairs with the filter before the key is changed for the second join operation, thus reducing the overall number of intermediate results.

#### Shuffle selection

The size of the first-joined RDD can be varied by joining the edge RDD or reverse-direction edge RDD. We choose fewer first-joined RDDs by choosing join operands based on the number of vertices of the sources and destinations of edges. We assume that an edge loaded from a file is

$$E=\{\left(v_{1},v_{2}\right)|v_{1},v_{2}\in V\}$$

A reverse-directed edge set can then be defined as follows:

$$R=\{\left(v_{2},v_{1}\right)|(v_{1},v_{2})\in E\}$$

We can define the set of join keys of E and R as follows:

$$K_{E}=\{v|(v,w)\in E\},K_{R}=\{v|(w,v)\in R\}$$

We can approximately calculate the number of KV pairs of first-joined RDDs with E and R. The number of KV pairs of first-joined RDDs is as follows:

$$R_{E}\approx \frac{\left|E\right|}{\left|K_{E}\right|}\times |E|,R_{R}\approx \frac{\left|R\right|}{\left|K_{R}\right|}\times |R|$$

From the above two equations, we can determine the result of the join operation, which depends on the number of distinct join keys. We simply calculate the number of distinct source vertexes and destination vertexes of each edge RDD. The join operation uses the reverse-direction edge RDD if the number of distinct source vertexes of the edge RDD is less than that of the destination vertex to reduce shuffling.

### The time complexity and space complexity of CASS

In this section, we analyze the time complexity and space complexity of CASS. To analyze the complexity of CASS, we divided our algorithm into three steps which consists of 1) calculating structure similarity, 2) reducing network, and 3) finding connected components.

#### The time complexity of CASS

When the number of nodes is M and the number of edges is N, the total time complexity of the proposed algorithm is as follows. First, the process of calculating structure similarity includes two join processes between edge data and the creation and search process of the Bloom filter. The two join processes to the edge have a time complexity of O(N^3^) when the network is fully connected in the worst case, and the time complexity of the Bloom filter generation and search are O(N) in the worst case. Next, in order to perform the reducing network process, the time complexity is O(N), because the structure similarity must be confirmed for all the edges. Finally, the process of finding connected components requires visiting the entire node and edge once, and therefore, the time complexity is (N + M). Since N is larger than M-1, the total time complexity is O(N^3^)

#### The space complexity of CASS

Similarly, when the number of nodes is *M* and the number of edges is *N*, the total space complexity of the proposed algorithm is as follows. First, in the process of obtaining structure similarity, we need two join operations. In the worst case, if the network is fully connected network, the first join operation needs O(N^2^) space complexity. This is because the first join operation approximatively generates nC2 candidate triangles which have two edges and three nodes. Next, the second join operation requires less space complexity than O(N^2^). The reason is that the triangle set found in the second join operation is a subset of the candidate triangle set. For example, when the case of an n-star network which is a tree on n nodes with one node having a vertex degree n-1 and the other n-1 having a vertex degree 1, the result of the second join operation is an empty set. Therefore, in the worst case, the space complexity of O(N^2^) is required to calculate structure similarity. In addition, the space complexity of the Bloom filter generation and search are O(N) in the worst case. Next, the space complexity of the reduction network process is O(N), because it needs the entire structure similarity of edges. Finally, in order to find the connected component, the refined node and edge information from the previous step is needed, so the space complexity is O (N + M). In sum, the total space complexity is O(N^2^) because N is larger than M-1.

## Results and discussion

In this section, we introduce experimental results to validate the performance of our proposed algorithm. To validate the proposed method, we compared the proposed algorithm with comparison algorithms. First, we compared the average normalized cut and the running time using five datasets. Second, we evaluated the performance of relative speedup and reuse time of structure similarity. Third, we verified our optimization effects by running time comparison experiments. Finally, we used the Humannet data to confirm that the clusters found from CASS have biologically significant functions.

### Experimental setting

We evaluate our algorithm on a cluster of five computers, each equipped with 64 GB of volatile memory and a quad-core 4.0 GHz processor. We use four computers as slave nodes and one computer as the master node. The algorithm has been implemented using Apache Spark 1.6.0. Hadoop 2.7.2 and Scala 2.10 were used in the experiments. The environmental variables and term definitions are presented in two tables. Table 2 outlines the environmental parameters and Table 3 presents the definitions of the terms used in the experiments.

**Table 2:** Environmental variables.

Parameter	Value	Description
Epsilon	0.7	Structure similarity threshold
False positive rate	0.3	False-Positive of Bloom filter
#{executors}	20	The number of executors for Spark jobs
# {data partitions}	20	The number of data partitions

**Table 3:** Term definitions.

Term	Definition
Running Time	Algorithm execution time
Reuse Time	Algorithm execution time by using calculated structure similarity
Avg N-cut	Average normalized cuts between clusters.
Relative speedup	Relative speedup of Algorithm execution time

As shown in Table 2, “Epsilon” was used as the threshold for the structure similarity. The false-positive rate was the probability that the output of the Bloom filter would be a false-positive. #{executors} was the number of executors of Spark, which performed transformations and actions in our algorithm. Data, which were processed by executors, were divided into #{data partitions}. We allotted 20 for #{data partitions}. In our experimental settings, each executor processed one data partition. Table 3 shows the terms used in the experimental results. “Running Time” indicates the general execution time, and “Reuse Time” indicates the execution time when reusing the structure similarity, which is already calculated.

Although environmental variables were changed, the structure similarity value remained the same. Therefore, we could reuse the structure similarity after the first algorithm execution process. In addition, “Avg N-cut” is the average of the normalized cut value between clusters. The normalized cut value is calculated as the following:

$$Ncut(A,B)=\frac{edge(A,B)}{edge(A)}+\frac{edge(A,B)}{edge(B)}$$

Above, “edge (A)” is the number of edges for nodes in cluster A. “Edge (A, B)” indicates the number of edges between clusters A and B. Finally, “relative speedup” refers to the speed increase according to the number of computers used in the algorithm. The relative speedup value is calculated as the following:

$$Relative\;speedup(k)=\frac{The\;execution\;time\;on\;2\;computers}{The\;execution\;time\;on\;k\;computers}$$

### Comparison experiments

In this section, we compare the proposed algorithm with existing network clustering algorithms to verify the performance of the proposed method. The algorithms that we chose to compare CASS with are MCL, PSCAN, k-center and SCAN++ clustering algorithms. The PSCAN and k-center algorithms are developed based on distributed systems which are the Hadoop MapReduce and The Apache Spark. The experiment environments are identical to that of our algorithm which consists of one name node and four data nodes. On the other hand, MCL and SCAN++ are each developed based on a single machine. Therefore, we use one computer out of the five computers to evaluate MCL and SCAN++.

**Fig 5:**
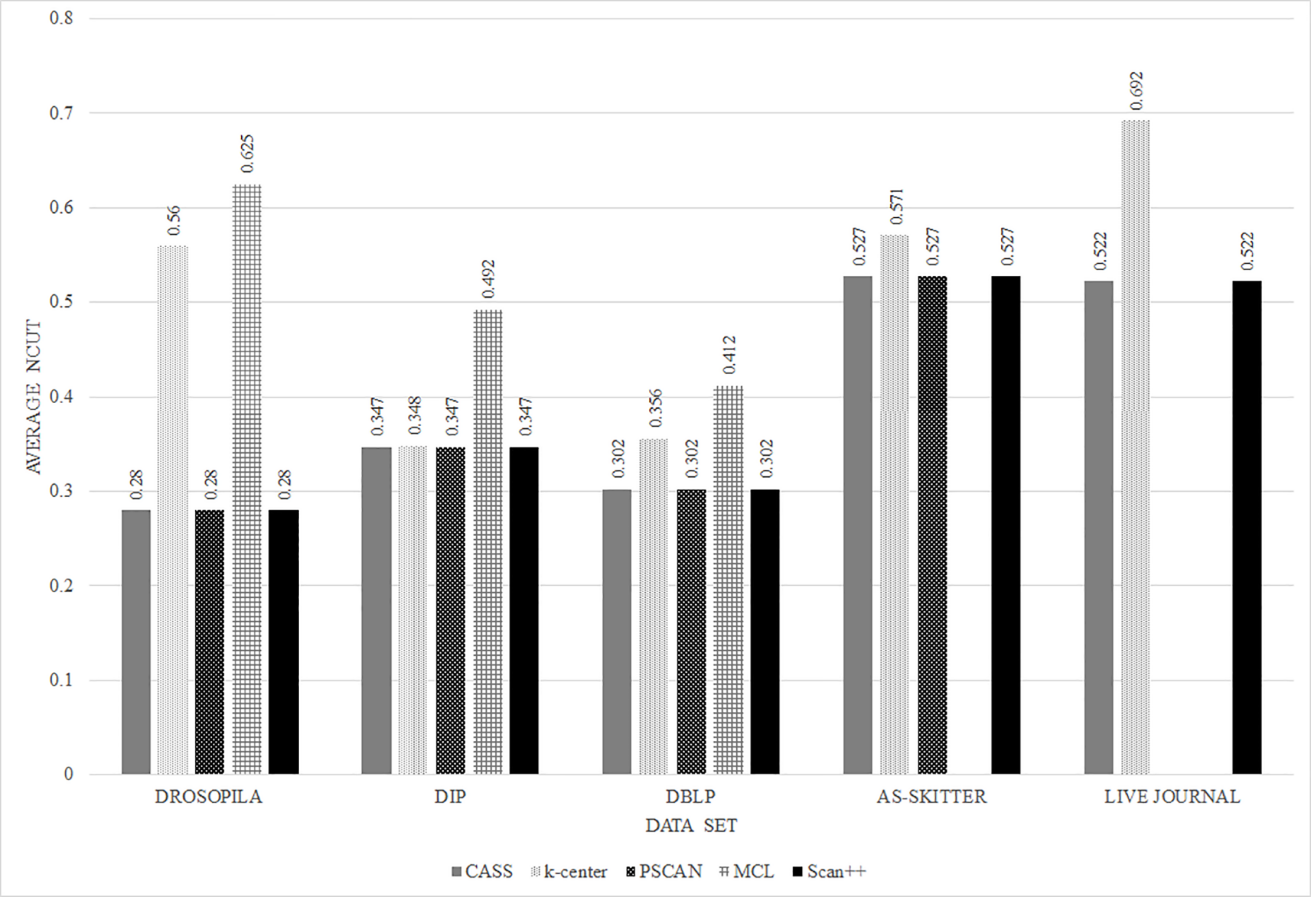
Results of the average normalized cut experiment.

First, we measure the quality of clusters found from each algorithm by calculating the average normalized cut. Note that the result of the k-center algorithm includes clusters which have only one node, and we exclude these clusters from the experiment. In the case of the k-center algorithm, we set the parameters of k-center algorithm to find almost the same number of clusters to the proposed method. Fig 5 shows the average normalized cut of clustering results for the proposed, MCL, PSCAN, k-center and SCAN++ clustering algorithms. The x-axis represents datasets and the y-axis represents average normalized cut. In this validation, the lower average normalized cut indicates better performance. As shown in Fig 5, the proposed algorithm has a lower value than the MCL and k-center algorithms for all datasets. Since PSCAN and SCAN++ look for clusters based on structure similarity, the result is the same as the proposed algorithm. These results indicate that the proposed algorithm locates clusters with higher quality than the MCL and k-center algorithms methods in the average normalized cut validation.

**Fig 6:**
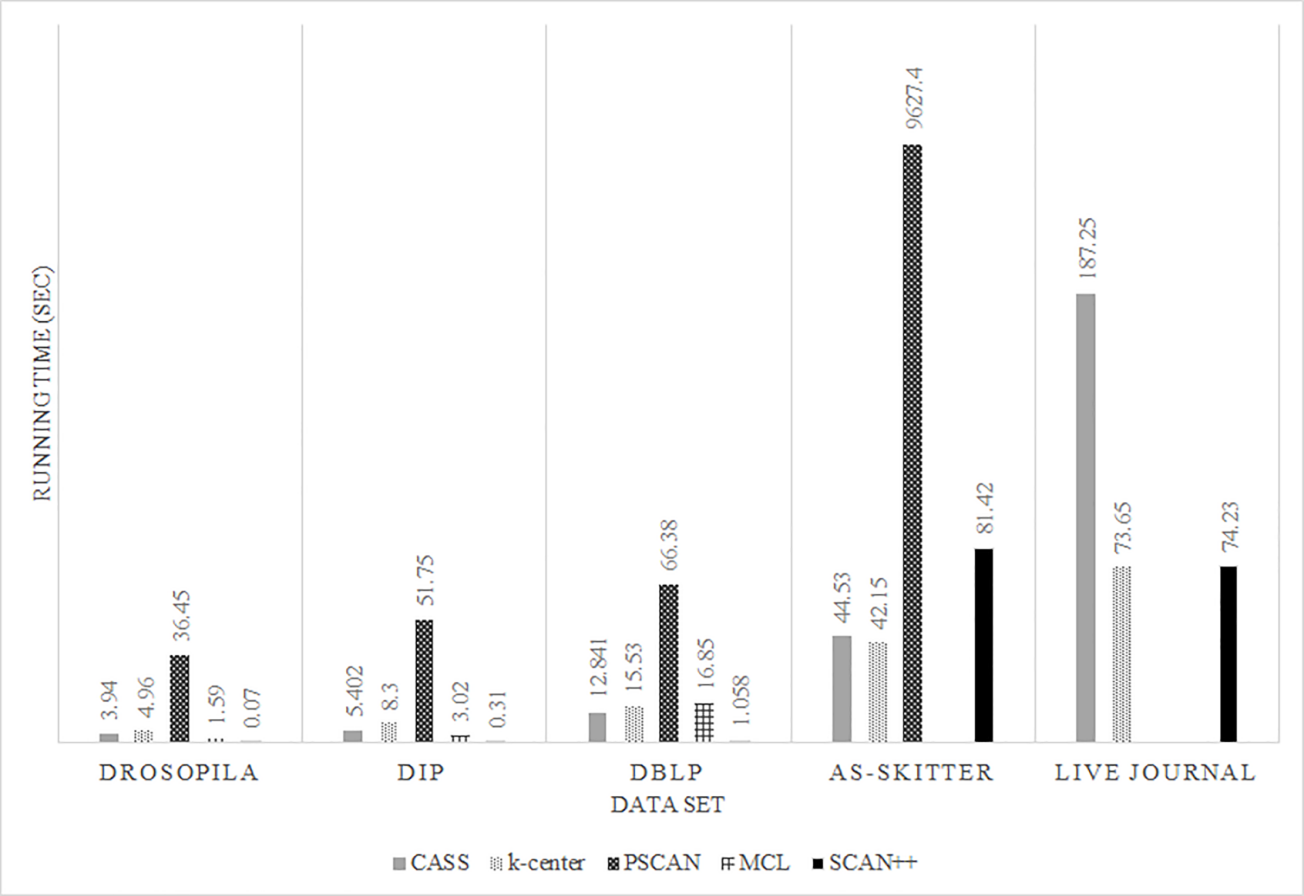
Results of the running time experiment.

We also evaluate the running time of the proposed, MCL, PSCAN, k-center and SCAN++ clustering algorithms. Fig 6 shows the experimental results. The x-axis represents datasets and the y-axis represents running time. The y-axis is rescaled using the log function. In small datasets such as DIP and Drosophila, SCAN++ is faster than the proposed method, because the proposed method requires partitioning and collection time to handle distributed computation. Therefore, if the size of the dataset is small, the proposed method is slower than SCAN++. However, if the data set is large enough, the speed difference between the proposed method and SCAN ++ is reduced. From this result, it may seem like the proposed algorithm is not as good as SCAN ++, but what is significant to note is that SCAN ++ is not an algorithm developed in a distributed system environment. Therefore, there is a critical limitation to further improving the speed. On the other hand, the proposed algorithm does not have this limitation because it was developed in a distributed system environment, which means that through CASS, increase in speed becomes possible. In the case of AS-SKITTER and LIVE JOURNAL, k-center is faster than the proposed method. The proposed algorithm includes the process to calculate edge weights based on structure similarity, and this process is affected by the average degree of the network nodes. On the other hand, since the k-center has no process to calculate edge weights, the higher the average degree of the network, the faster it performs compared to the proposed algorithm. However, as shown in Fig 5, the proposed algorithm calculates edge weights and uses them to enable more accurate network analysis than k-centers. In addition, we also confirm that MCL cannot analyze AS-SKITTER and LIVE JOURNAL data because of memory limitations and PSCAN cannot analyze LIVE JOURNAL data. Also, in the case of AS-SKITTER, it was confirmed that the speed of the PSCAN decreases significantly.

**Fig 7:**
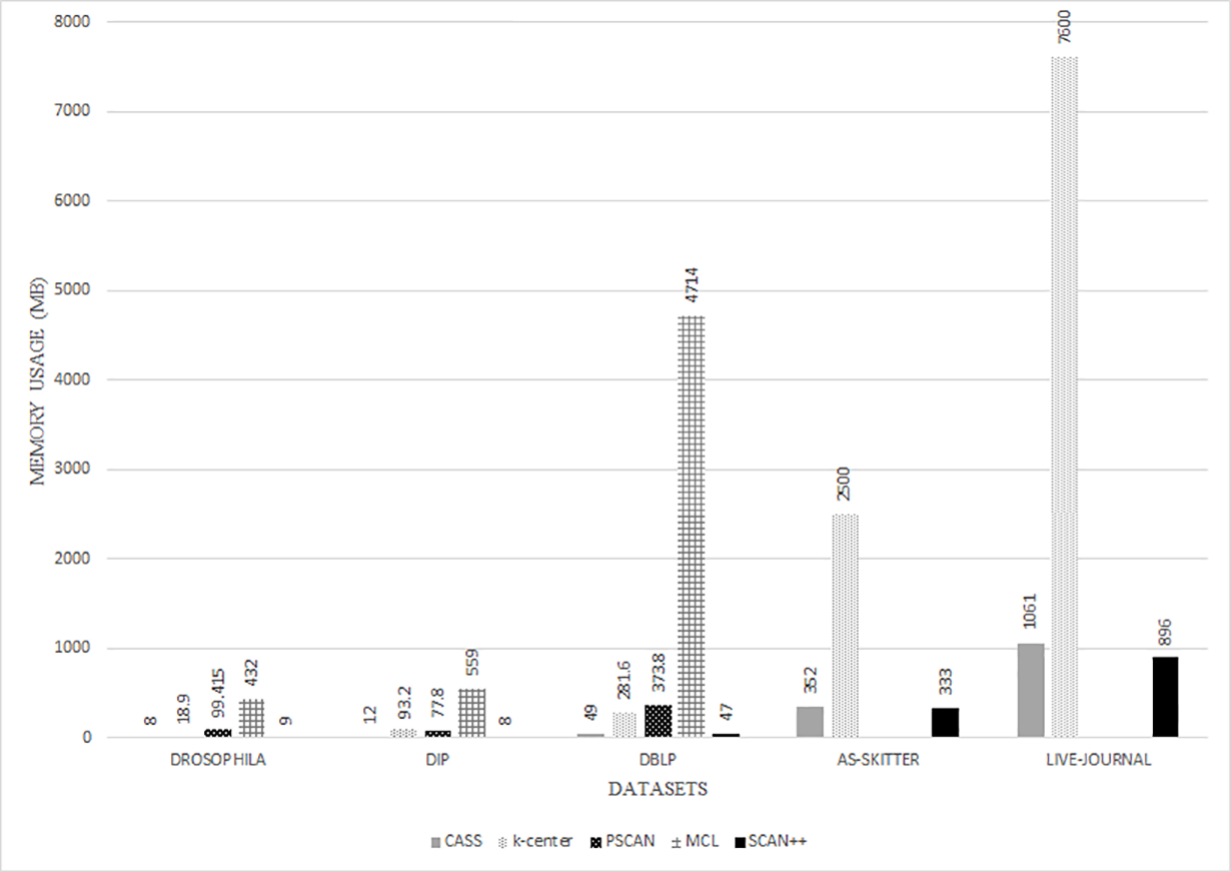
Results of the total memory usage.

**Fig 8:**
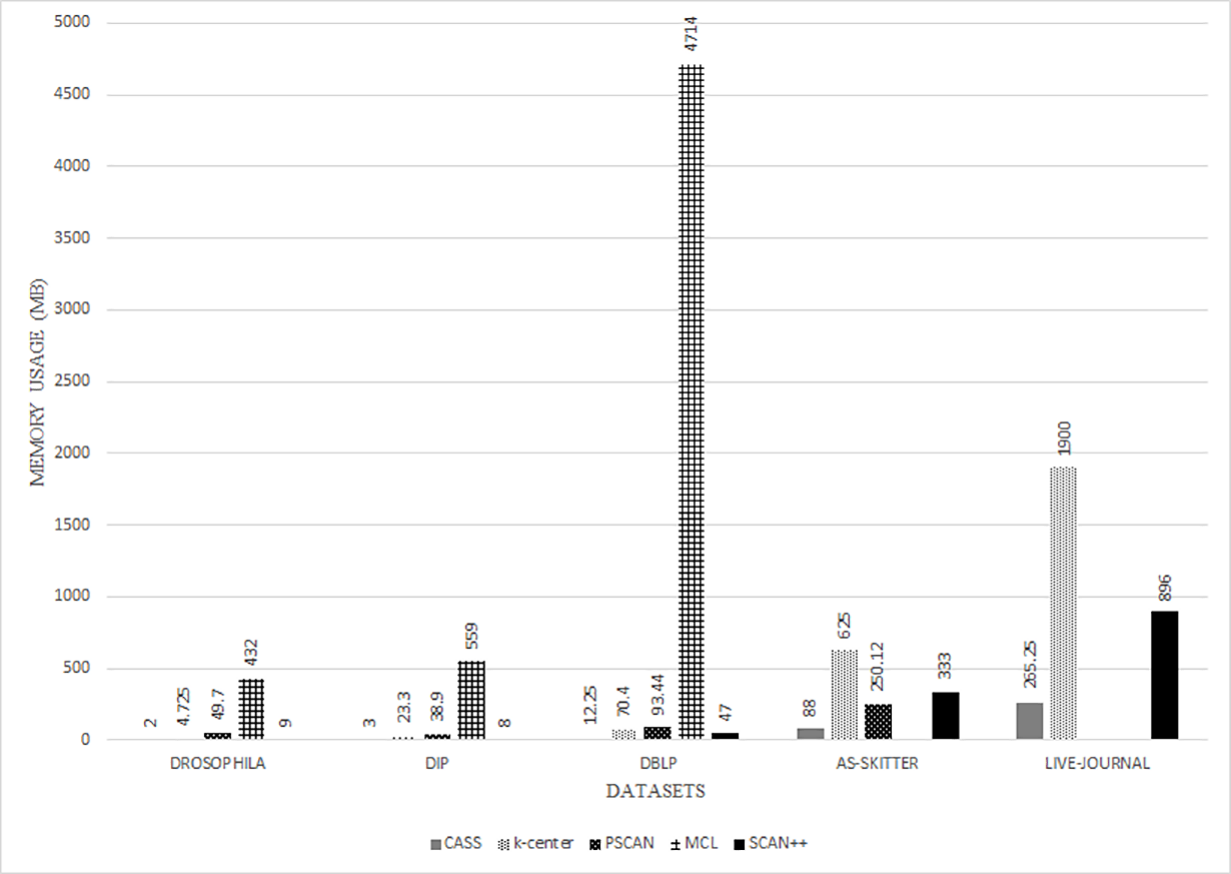
Results of the average memory usage.

Finally, we calculated the memory usage of all the algorithms. Fig 7 shows the total memory usage needed to analyze each dataset, and Fig 8 shows the average memory usage needed per computer. For both figures, the x-axis represents the datasets and the y-axis represents memory usage. As shown in Fig 7, MCL, k-center, and PSCAN required more than two times the memory used for our proposed algorithm. Although the total memory usage of CASS was slightly more than SCAN++, as shown in Fig 8, the memory usage of CASS for one computer was less compared to SCAN++. Since CASS was developed in a distributed system environment, the total memory usage is divided by the total number of computers.

### Performance of re-use time and relative speedup

**Fig 9:**
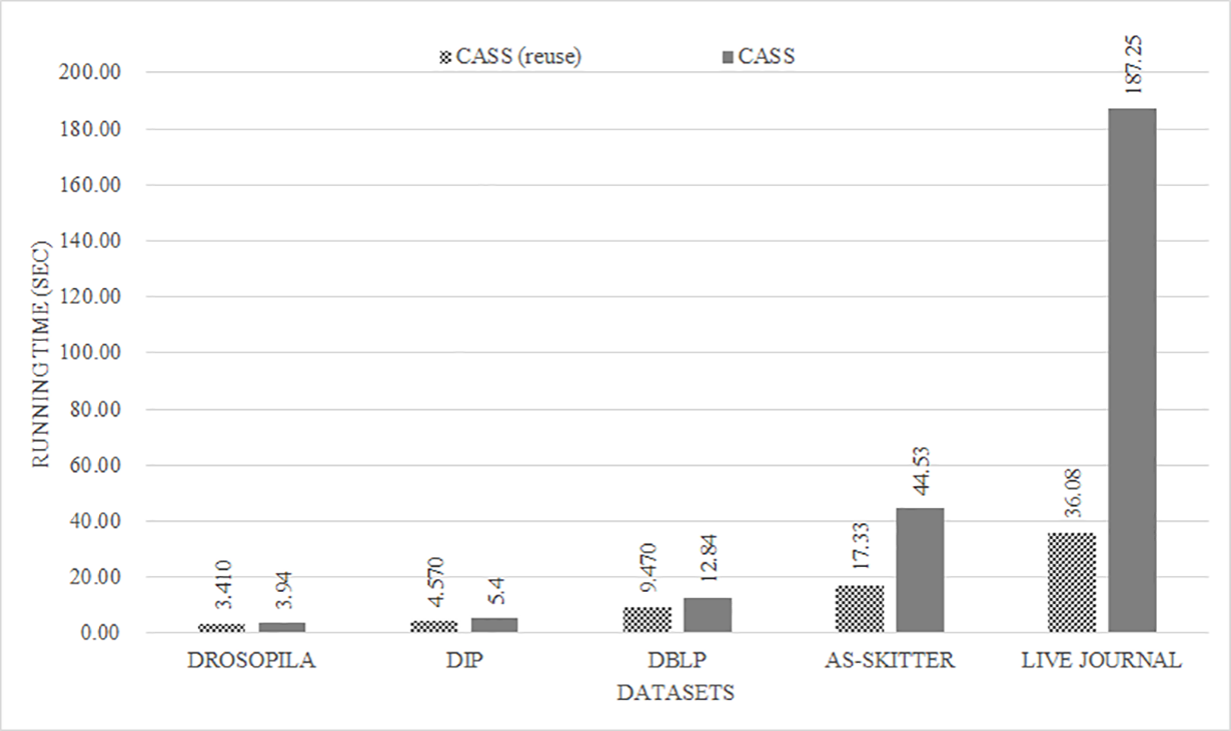
Results of the reuse time for CASS.

The proposed algorithm can store and reuse the calculated structure similarity. Fig 9 shows the running time when the calculated structure similarity is used. The x-axis represents datasets and the y-axis represents running time. For all cases, the running time is reduced. In the case of LIVE JOURNAL, the running time is reduced by approximately five times. From this result, we confirm that the proposed method is advantageous in an environment where the network must perform repeated analyses using various variables.

**Fig 10:**
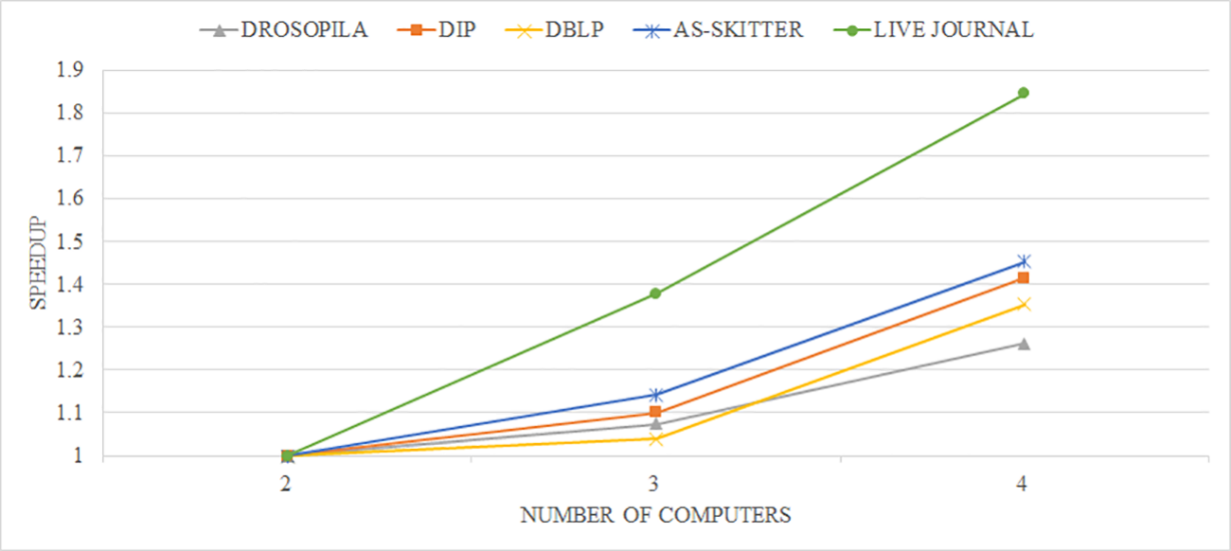
The relative speedup of CASS.

To evaluate the speedup, we measure the running time of the proposed algorithm when two, three, and four computers are used. Fig 10 shows the experimental results regarding relative speedup. The x-axis represents datasets and the y-axis represents relative speedup. As shown the figure, we confirmed that the proposed algorithm is very effective at relative speedup. In particular, note that for the large-scale network (LIVE JOURNAL), the relative speedup also increases more rapidly than for the smaller networks as the number of computers increases.

**Fig 11:**
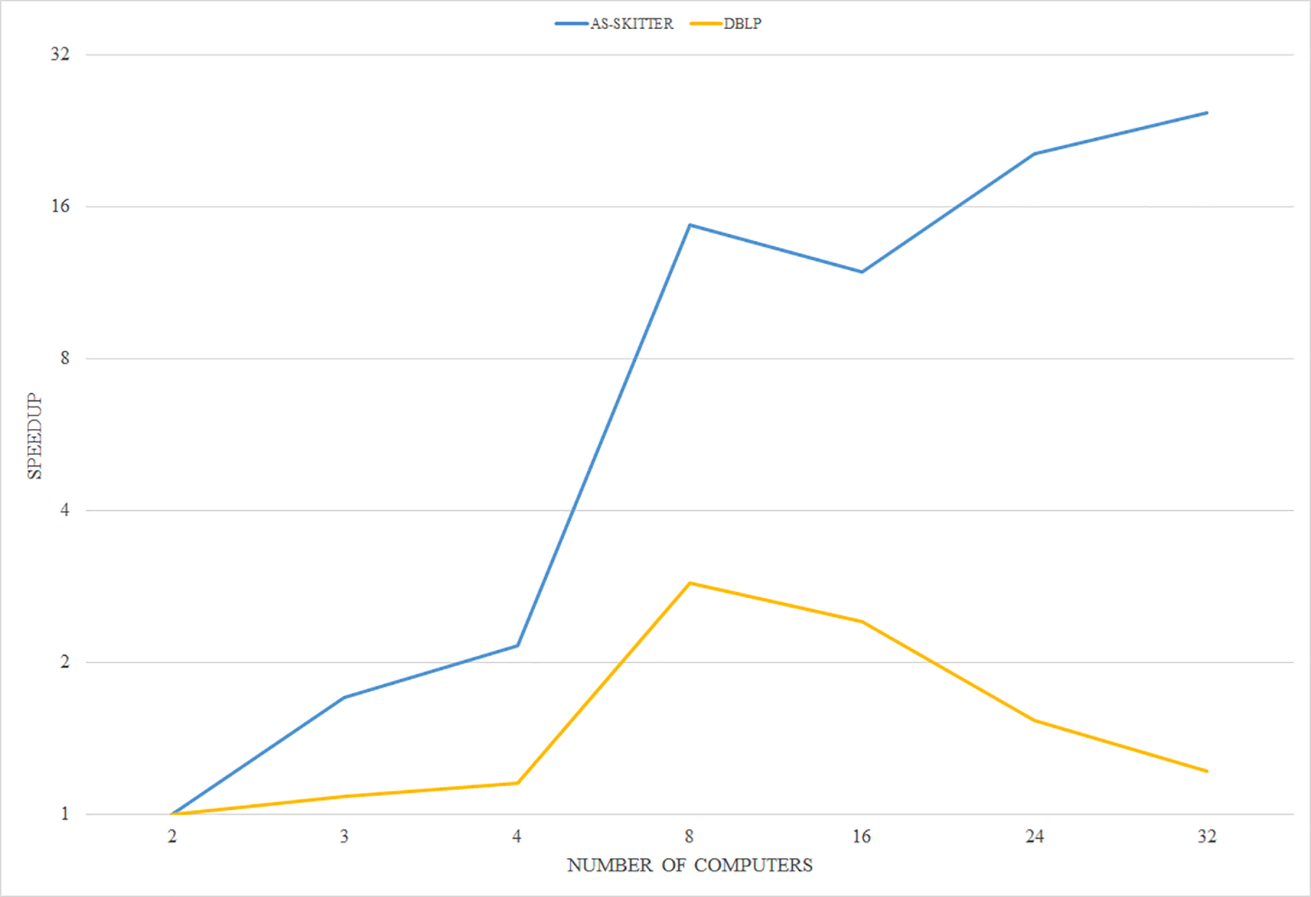
The relative speedup of CASS using amazon web services.

In addition, we confirmed how much speed improvement could be possible by increasing the number of nodes in the distributed system to 32 for AS-SKITTER and DBLP data. This experiment was performed on the computers with CPU 2.3GHz Intel Xeon E5-2686 v4 (Broadwell) or 2.4GHz Intel Xeon E5-2676 v3 (Haswell) processor, 4 vcores, 8 GB RAM. The experimental results are shown in Fig 11. In the case of AS-SKITTER data, when using 32 nodes, the performance was 25.5 times faster than using 2 nodes. On the other hand, DBLP has improved performance (up to 2.8 times) until the use of 8 nodes, but performance degradation was observed when using more nodes. It is judged that this is caused by analyzing small data unnecessarily at too many nodes.

### Performance of the optimization

To verify the optimization effects, we applied the proposed algorithm with and without optimizations. The differences between optimization and non-optimization are evaluated by considering various cases.

**Fig 12:**
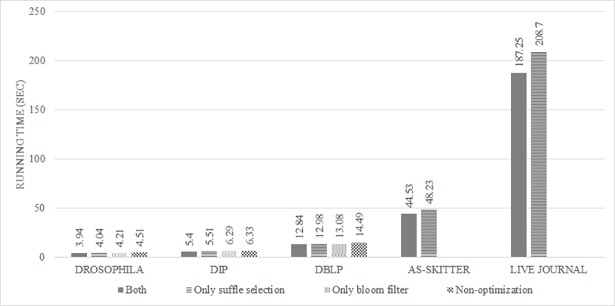
Shuffle selection and Bloom filter results for CASS.

Fig 12 shows the running time for four cases: (1) using both shuffle selection and Bloom filter, (2) using shuffle selection (3) using Bloom filter, and (4) no optimization. From the experiment result, the effect of the proposed optimization technical contributions may not seem to be significant for small network size data such as Drosophila, DIP and DBLP. The reason for this is that, as a distributed system platform feature, the cost of network for loading data basically takes up a relatively large portion as the dataset becomes smaller. In other words, when the data size is small, it appears as if there is little benefit of optimization. However, it is important to note that even with small data, a performance improvement of 12% or more is obtained when the proposed optimization is applied. Considering this fact, we confirmed that the running time was reduced by using shuffle selection and Bloom filter for all datasets. Furthermore, in the cases of AS-SKITTER and LIVE JOURNAL, we confirmed that the algorithm cannot cover the networks because of memory limitations when shuffle selection is excluded. These results show that the proposed optimizations are useful for reducing the running time and overcoming memory limitations.

### Parametric study

In this section, we performed experiments to confirm changes in the clustering results according to the values of m and epsilon, which are parameters used in the CASS algorithm. The experimental results are shown in Table 4. As shown in the table, we used m from 2 to 4 and epsilon from 0.3 to 0.7. In the table, average normalized cut is used in the same way as the previous experiment, and the cluster size shows the number of clusters found when the corresponding parameter is used. Next, the single node ratio shows the ratio of the remaining nodes not included in the resulting clusters. Experimental results show that the clustering results vary greatly according to the m value rather than the epsilon value. Also, when m is 4, it is confirmed that more than half of the nodes are not included in the resulting clusters for the data sets except DBLP.

**Table 4:** Result of a parametric study.

		Average normalized cut					The cluster size					The remaining node ratio				
	*eps /* *m*	*0*.*3*	*0*.*4*	*0*.*5*	*0*.*6*	*0*.*7*	*0*.*3*	*0*.*4*	*0*.*5*	*0*.*6*	*0*.*7*	*0*.*3*	*0*.*4*	*0*.*5*	*0*.*6*	*0*.*7*
**Dataset**																
**Drosophila**	2	0.58	0.49	0.38	0.27	0.25	586	360	124	30	5	0.5	0.79	0.93	0.98	0.99
	3	0.57	0.41	0.28	0.14	-	344	77	13	2	-	0.9	0.94	0.99	0.99	-
	4	0.51	0.37	0.09	-	-	154	20	1	-	-	0.87	0.98	0.99	-	-
**DIP**	2	0.52	0.42	0.36	0.35	0.34	1,430	1,558	1015	477	214	0.09	0.62	0.81	0.92	0.96
	3	0.49	0.4	0.38	0.43	0.46	1,212	832	390	142	51	0.57	0.78	0.91	0.97	0.99
	4	0.45	0.39	0.43	0.49	0.47	850	429	164	54	24	0.69	0.87	0.95	0.98	0.99
**DBLP**	2	0.21	0.29	0.35	0.38	0.4	3,652	10,714	23,600	31,157	30,745	0.07	0.16	0.28	0.4	0.54
	3	0.25	0.33	0.36	0.37	0.38	3,810	10,465	18,977	20,754	17,070	0.18	0.28	0.41	0.43	0.7
	4	0.31	0.38	0.38	0.38	0.37	4,201	10,736	15,080	13,528	9,623	0.29	0.38	0.54	0.68	0.8
**AS-SKITTER**	2	0.55	0.56	0.55	0.54	0.52	83,255	100,649	72,262	37,548	15,773	0.44	0.65	0.81	0.91	0.96
	3	0.62	0.62	0.62	0.61	0.61	63,810	56,355	27,337	10,037	3,246	0.62	0.8	0.92	0.97	0.99
	4	0.64	0.63	0.63	0.62	0.64	50,253	32,554	12,194	3,594	1,057	0.71	0.87	0.95	0.98	0.99
**LIVE-JOURNAL**	2	0.56	0.55	0.55	0.55	0.52	91836	173,601	183,598	130,727	74,247	0.34	0.57	0.78	0.86	0.89
	3	0.57	0.57	0.57	0.56	0.52	64,878	112,567	105,224	65,659	32,752	0.48	0.71	0.91	0.93	0.95
	4	0.57	0.57	0.58	0.56	0.5	48,460	80,488	67,976	38,537	18,257	0.58	0.81	0.95	0.95	0.98

### Analysis of running time and memory usage according to network size

**Fig 13:**
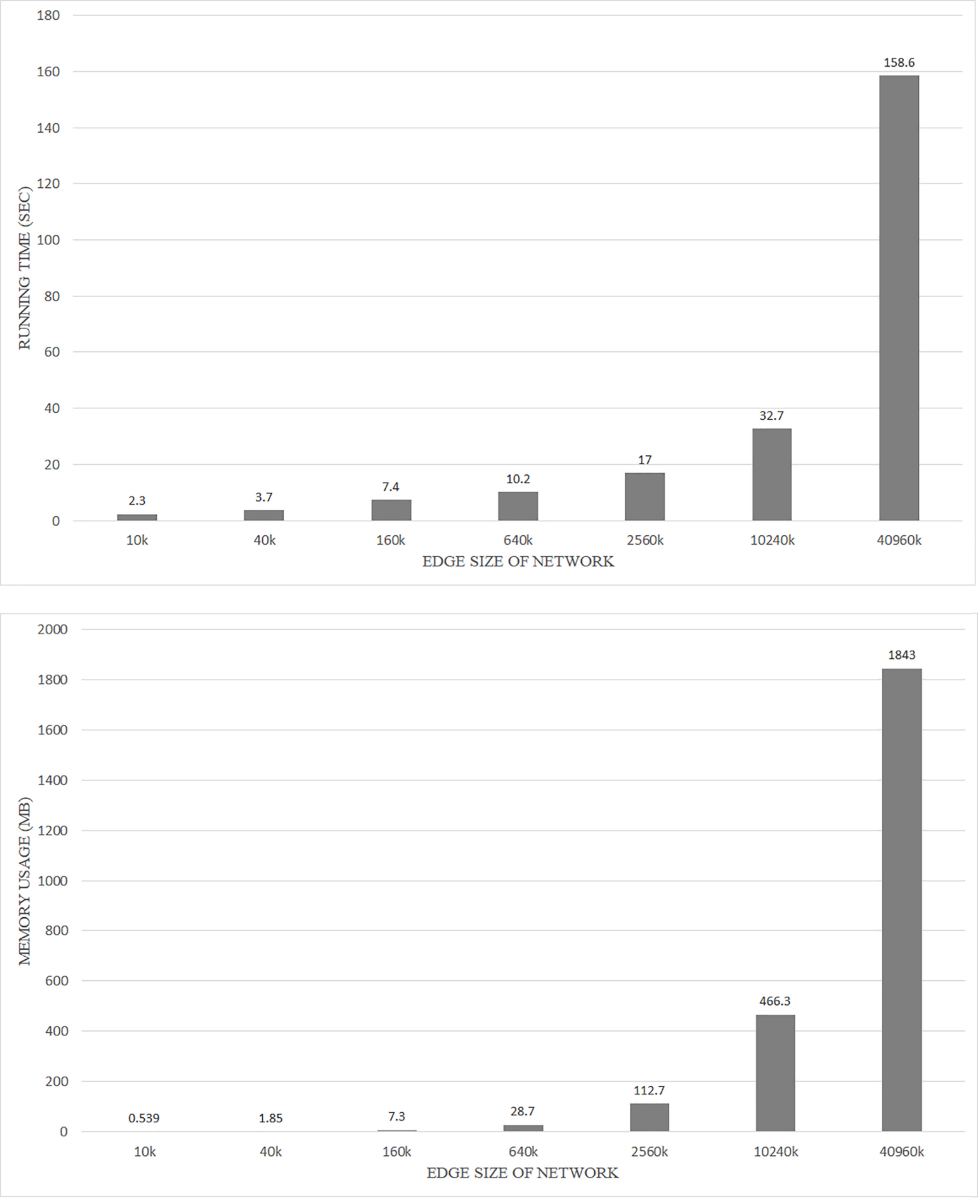
The result of running time and memory usage by network size.

In this section, we conducted an experiment to present a guideline of the running time and memory usage the proposed method requires depending on network size. For the experiment, we used the Erdos Renyi model to generate a random network [24] with a degree of 20 and an edge size of 10,000 to 40,960,000. In order to measure the running time and memory usage, we used four computers. To check the memory usage, we used the average of the amount of memory used in each computer. The experimental results are shown in Fig 13. In the figure, the x-axis represents the edge size of the network and the y-axis represents running time and memory usage, respectively. The results show that the size of the network increases and the variation of running time and memory usage tends to increase as the network size increases.

### Analysis of Humannet

In this section, we discuss the validation of the clusters obtained from the proposed algorithm. In other words, we analyzed Humannet to see if the proposed algorithm could be used to analyze biological networks. Humannet is a genetic network constructed using meaningful genetic interaction data from 21 different organisms for revealing human gene function. Therefore, each node (gene) is involved not only in a specific function but also in various functions at the same time. In addition, interactions between genes are involved in the same function. To validate clusters of Humannet, we compared clusters with the Kyoto Encyclopedia of Genes and Genomes (KEGG) pathway and the Gene Ontology (GO) term. The KEGG pathway is a collection of networks, which represents molecular interactions and reactions related to biological processes such as cellular processes, metabolism, and human diseases. The GO term is a collection of gene sets that have the same functions along with three aspects: 1) biological processes, 2) cellular components, and 3) molecular functions. For KEGG pathway analysis, we used the Database for Annotation, Visualization and Integrated Discovery (DAVID) v6.8 [25,26], which provides functional annotation tools to detect biological meaning using a gene list. To identify GO terms that are shared with clusters, we used the FuncAssociate tool [27], a GO term enrichment algorithm, which uses gene lists as the input and outputs the enriched GO terms that the gene module shares, along with the p-value. For the experiment, we analyzed Humannet v.1, which is a probabilistic functional gene network of homo sapiens. Among these clusters, 66 clusters were significantly related to KEGG pathways and 175 clusters were significantly related to GO terms. Detailed results and cluster information are presented in S1 File. From this result, we confirmed that the proposed algorithm has good performance for finding meaningful gene modules from biological networks. In addition, we measured the average normalized cut and the modularity which measure the strength of division of the clusters from Humannet, which were 0.714 and 0.125, respectively.

**Fig 14:**
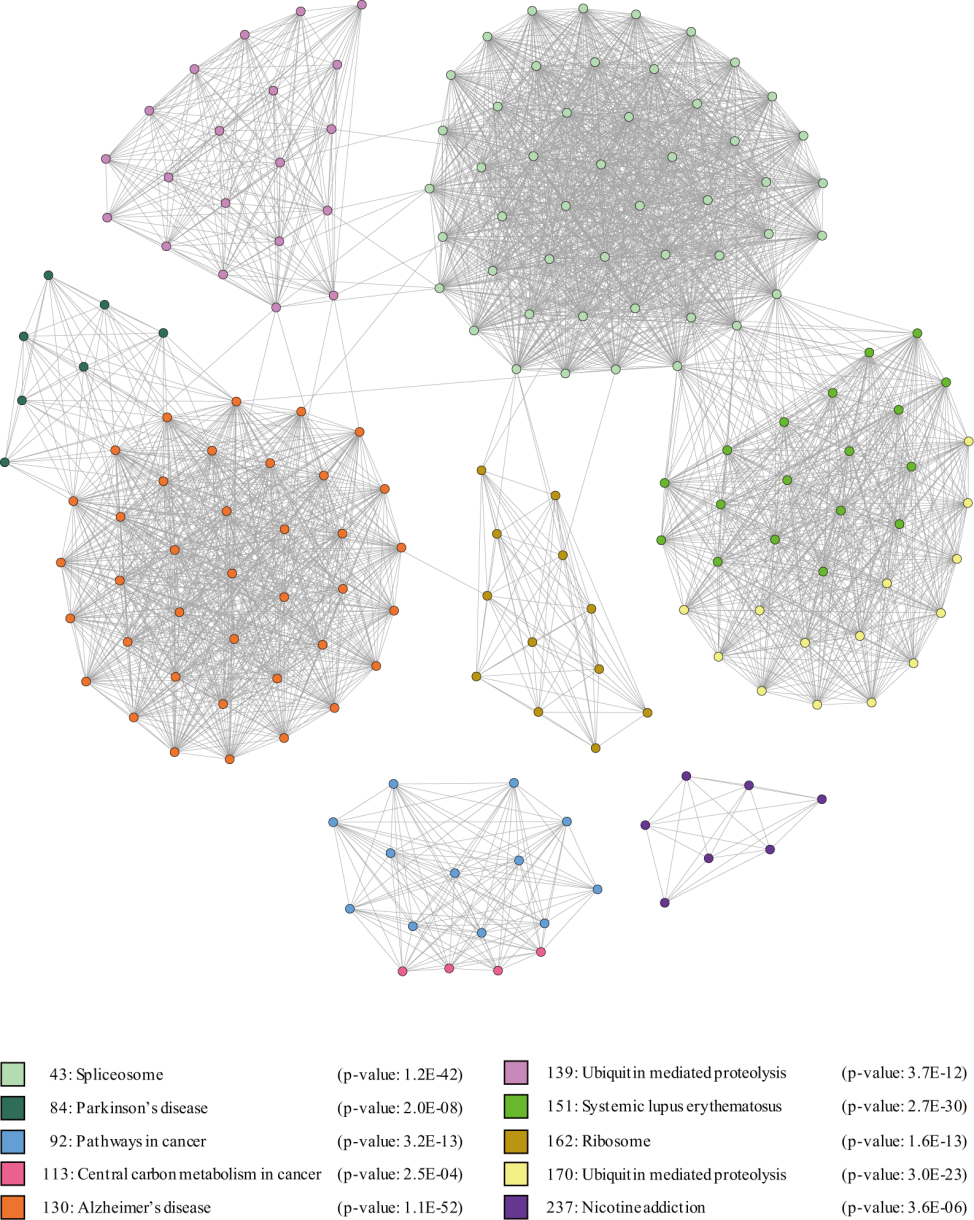
The KEGG pathway enrichment experiment result.

In addition, we further analyzed the relationship among the obtained clusters. To do this, we constructed a sub-network consisting of 10 clusters that were statistically related to the KEGG pathway. Then, we analyzed the relationship between the clusters closely connected by edges. Fig 14 shows the clusters described above. In this figure, the number shown next to the square box indicates the cluster id and, the name of the pathway shown next to the cluster id indicates the KEGG pathway that has a statistically relationship with the cluster and the p-value; for example, “43: Spliceosome (p-value: 1.2E-42)” indicates that cluster 43 is statistically related with the Spliceosome KEGG pathway with a p-value of 1.2E-42. As shown in Fig 14, there are closely connected clusters by edges such as clusters 139 and 49. To confirm the relationships between these clusters, we conducted a case study. Table 5 represents the research that revealed the relationships between cluster A and cluster B. Here, for example, the first row shows that a research on ubiquitin mediated proteolysis, which is closely related to cluster 139, is significantly associated with spliceosome, which is closely related to cluster 43.

**Table 5:** Results of a case study.

Cluster A	Cluster B	Summary	Reference
139	43	Ubiquitin mediated proteolysis is significantly related with spliceosome	[28]
139	130	Dysfunction of ubiquitin-mediated proteolysis is related with Alzheimer's disease	[29]
139	84	Dysfunction of ubiquitin-mediated proteolysis is related with Parkinson's disease	[29]
130	84	Alzheimer's disease and Parkinson's disease are both neurodegenerative diseases, closely related to each other	[30], [31]
130	162	Dysfunction of ribosome is an initial event of Alzheimer's disease	[32]
43	151	Systemic lupus erythematosus is closely related to the essential component of spliceosome, snRNP	[33]

As shown in the results of the case study, we confirmed that clusters closely connected to each other on the network have significant relationships.

## Conclusion

In this paper, we proposed a distributed network Clustering Algorithm based on Structure Similarity (CASS) that can analyze large-scale undirected networks using Apache Spark. (The CASS algorithm can be downloaded and used through the following link: https://github.com/Bluejung/CASS) To develop the algorithm for a distributed system, we changed the paradigm of the algorithm using join operations. We also optimized the proposed algorithm to reduce memory usage and execution time by using optimization approaches.

To verify the performance of the proposed method, we applied our algorithm to small networks as well as large-scale networks. Experimental results showed that the proposed algorithm can analyze large-scale networks, and demonstrated that the proposed algorithm locates clusters with higher quality than other algorithms. Although the proposed algorithm requires more time to analyze a large-scale densely connected network than the k-center algorithm, the proposed algorithm calculates the weights of the edges and uses these to enable more accurate network analysis in the densely connected case. Furthermore, we confirmed that proposed algorithm requires less memory usage than other comparison algorithms. Moreover, the proposed algorithm can save the result from calculating the structure similarity and reuse it, which is very advantageous in a situation where various analyses of the same network are required. In addition, we confirmed the scalability of the proposed algorithm through experiments and confirmed the effects of the optimizations involving shuffle selection and the Bloom filter. We also validated that the proposed algorithm can find clusters that can represent biologically meaningful functions using Humannet.

In future works, we plan to develop a network clustering algorithm for directed large-scale networks. In addition, because there have been few network clustering algorithms developed for distributed systems that consider the characteristics of social networks, we also plan to develop a network clustering algorithm for large-scale social networks.

## Supporting information

S1 File The information of clusters. This file includes the information of clusters which is result of Analysis of Humannet.
(XLSX)
